# Prognostic Utility of the Modified Glasgow Prognostic Score in Urothelial Carcinoma: Outcomes from a Pooled Analysis

**DOI:** 10.3390/jcm11216261

**Published:** 2022-10-24

**Authors:** Daqing Tan, Jinze Li, Tianhai Lin, Ping Tan, Jiapeng Zhang, Qiao Xiong, Jinjiang Jiang, Yifan Li, Peng Zhang, Qiang Wei

**Affiliations:** 1 Department of Urology, Institute of Urology, West China Hospital, Sichuan University, Chengdu 610041, China; 2 Department of Urology, Minda Hospital of Hubei Minzu University, Enshi 445000, China

**Keywords:** modified Glasgow Prognostic Score, urothelial carcinoma, prognosis, survival, meta-analysis

## Abstract

Background: Many studies explored the prognostic value of the modified Glasgow Prognostic Score (mGPS) in urothelial carcinoma (UC), but the results are controversial. This study aimed to quantify the relationship between pretreatment mGPS and survival in patients with UC. Methods: A systematic literature search was conducted using Embase, PubMed, and Web of Science to identify eligible studies published before August 2022. Pooled hazard ratios (HRs) with 95% confidence intervals (CIs) were used to assess the association between pretreatment mGPS and the prognosis of UC. Results: Thirteen eligible studies involving 12,524 patients were included. A high mGPS was significantly associated with poor overall survival (mGPS 1/0: HR = 1.33, 95% CI 1.12–1.58, *p* = 0.001; mGPS 2/0: HR = 2.02, 95% CI 1.43–2.84, *p* < 0.0001), progression-free survival (mGPS 1/0: HR = 1.26, 95% CI 1.03–1.53, *p* = 0.021; mGPS 2/0: HR = 1.76, 95% CI 1.12–2.77, *p* = 0.013), recurrence-free survival (mGPS 1/0: HR = 1.36, 95% CI 1.18–1.56, *p* < 0.0001; mGPS 2/0: HR = 1.70, 95% CI 1.44–2.000, *p* < 0.0001), and cancer-specific survival (mGPS 2/0: HR = 1.81, 95% CI 1.30–2.52, *p* < 0.0001). A subgroup analysis of OS also yielded similar results. Conclusions: Evidence suggests that high pretreatment mGPS in UC is closely related to poor survival. Pre-treatment mGPS is a powerful independent prognostic factor in patients with UC.

## 1. Introduction

Urothelial carcinoma (UC), including bladder cancer (BC) and upper urinary tract urothelial carcinoma (UTUC), is a common tumor of the urinary system. More than 90% of bladder cancer is histologically classified as UC [1]. UTUC is relatively rare, accounting for only 5–10% of all UCs [2]. Due to the multifocal nature of UC throughout the entire urinary tract (synchronously or metachronously), the 5-year survival rate is only 50% even after radical resection of BC, and 15–50% of UTUC patients undergoing surgical treatment experience recurrence during follow-up [3,4,5]. Therefore, a simple and accurate indicator is urgently needed for the early detection and identification of progression or prognosis in patients with UC.

The current prognostic prediction models mostly rely on clinicopathological features obtained from retrospective analysis [6,7]. However, studies have found that the prediction accuracy of these models is limited [8,9,10]. As a non-invasive method, a blood-based biomarker analysis is more attractive for various cancers [11,12]. At the same time, much evidence suggests that clinical factors alone are not sufficient to predict the survival rate of patients with UC, and the systemic inflammatory response and nutritional deficiency might play a vital role in the development and progression of cancer [13]. The modified Glasgow Prognostic Score (mGPS) (Table 1) is a combination of C-reactive protein (CRP) and albumin, reflecting the inflammation and nutritional status of patients, and has an independent prognostic value for patients with various cancers, such as liver, lung, and colon cancer [14,15,16].

Many studies have shown that the mGPS has an important predictive value in the treatment of UC. However, due to differences in treatment methods and tumor staging, the results are inconsistent [17]. It is necessary to evaluate the prognostic value of the mGPS in patients with UC using a pooled analysis. Meta-analyses provide more reliable and accurate estimates of outcomes than individual studies. The aim of this study was to evaluate the relationship between pretreatment mGPS and the survival of patients with UC.

## 2. Materials and Methods

### 2.1. Protocol

This study followed the 2020 Preferred Reporting Items for Systematic Reviews and Meta-Analyses (PRISMA) guideline [18] and was registered in PROSPERO (CRD42022356946).

### 2.2. Literature Search Strategy

We searched the literature in Embase, PubMed, and the Web of Science from inception to August 2022. The following search terms were used for literature retrieval: (upper urinary tract urothelial carcinoma OR upper urinary tract carcinoma OR upper-tract urothelial carcinoma OR UTUC), (bladder cancer OR bladder neoplasms OR bladder tumor), and (Glasgow prognostic score OR GPS). To avoid missing literature, we searched for a list of references to relevant reviews and meta-analyses. Differences were resolved through discussion or by third-party ruling.

### 2.3. Inclusion/Exclusion Criteria

The inclusion criteria for eligible studies were as follows: (1) patients were diagnosed with UC by histopathology; (2) research aimed at studying the relationship between mGPS and survival results in patients with UC, such as overall survival (OS), progression-free survival (PFS), recurrence-free survival (RFS), and/or cancer-specific survival (CSS); (3) the hazard ratio (HR) and 95% confidence interval (95% CI) of survival results were reported; (4) studies published in English as full-text articles; (5) mGPS scores were computed before treatment. The exclusion criteria were as follows: (1) repetitive articles; (2) experimental or non-human studies; (3) studies focusing on the relationship between GPS and survival outcomes in patients with UC; (4) reviews, editorials, case reports, letters, comments, meta-analyses, and conference abstracts; and (5) incomplete or unavailable data. 

### 2.4. Data Extraction and Quality Assessment

Two researchers (D.T. and J.L.) independently extracted data from the eligible studies, and any differences between the two investigators were resolved via discussions or by a third-party decision. The following data were extracted from each study: first author, study area, publication year, sample size, research design, tumor type, tumor stage, patient age, survival outcome parameters, treatment strategy, and average follow-up. All survival results were directly expressed as HR and the corresponding 95% CI. When the data in the study were analyzed in both univariate and multivariate analyses, multivariate analysis data were used. The Newcastle–Ottawa Scale (NOS) was used to assess the quality of the included studies [19]. In this meta-analysis, studies were considered to be of high quality when the score was ≥7. The risk of bias of included studies was assessed using the Quality In Prognosis Studies (QUIPS) tool [20].

### 2.5. Statistical Analysis

All statistical analyses were performed using STATA v.14 (StataCorp, College Station, TX, USA). Pooled HRs with corresponding 95% CIs were used to assess the association between the mGPS and survival results. Heterogeneity among studies was evaluated using Cochran’s Q and Higgins I^2^ tests. I^2^ > 50% or *p* < 0.10 noted significant heterogeneity. This meta-analysis used the random-effects model for summary analysis. A subgroup analysis of the primary survival outcome, OS, was conducted to explore the potential sources of heterogeneity. A sensitivity analysis was also conducted to assess the impact of individual research data on survival outcomes. HRs and 95% CIs were used to assess the relationship between mGPS and survival outcomes in UC. We did not evaluate publication bias because fewer than 10 available studies were not convincing [21].

## 3. Results

### 3.1. Study Selection

**Figure 1:**
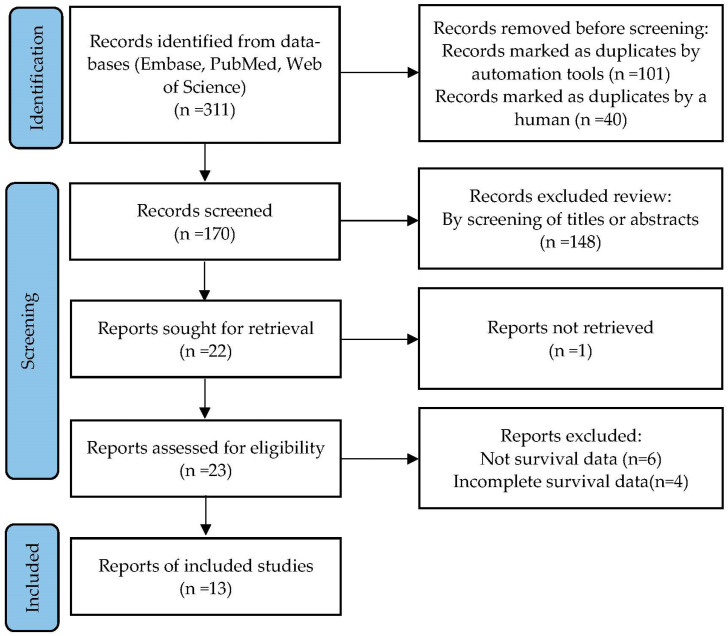
Flow diagram of literature search.

A total of 311 papers were retrieved from Embase, PubMed, and Web of Science databases. According to the PRISMA guidelines, a flow chart of the literature selection process is shown in Figure 1. After excluding unqualified studies, 13 studies including 12,524 patients were included in this pooled analysis [17,22,23,24,25,26,27,28,29,30,31,32,33]. 

### 3.2. Study Characteristics

**Figure 2:**
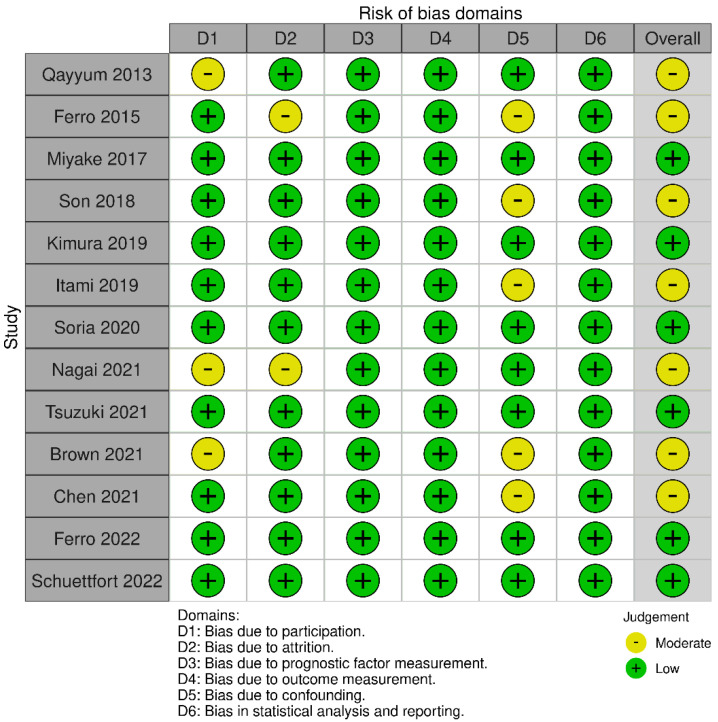
Risk of bias using the Quality in Prognosis Studies tool [20,34]. Low risk of bias; Moderate risk of bias [17,22,23,24,25,26,27,28,29,30,31,32,33].

The main characteristics of the 13 included studies are presented in Table 2. All included studies were retrospective analyses and were published from 2013 to 2022. Seven studies were on BC [17,22,23,24,30,31,33], four studies were on UTUC [25,26,27,28], and two studies were on both BC and UTUC [29,32]. Six studies were conducted in Asia (China, Japan, and Korea) [23,25,26,28,30,32], and seven studies were conducted in Western countries (the United States, Italy, the United Kingdom, and Austria) [17,22,24,27,29,31,33]. The treatment methods include surgical treatment, immunotherapy, and chemotherapy. The sample size of the study ranged from 53 to 4335, and the median age of the patients ranged from 67 to 72 years. Eight studies reported a correlation between mGPS and OS [17,23,26,27,28,29,30,31], seven investigated the associations between mGPS and CSS [17,22,25,27,28,31,32], seven investigated the associations between mGPS and RFS [17,24,25,27,28,31,33], and five reported an association between mGPS and PFS [24,29,30,32,33]. Except for one study that only included evaluation data in the univariate analysis [30], most studies used multivariate analysis for evaluation. All studies had NOS scores > 7, except for Nagai [32], indicating that the overall quality of the included studies was high. The bias assessment is shown in Figure 2.

### 3.3. mGPS and OS

**Figure 3:**
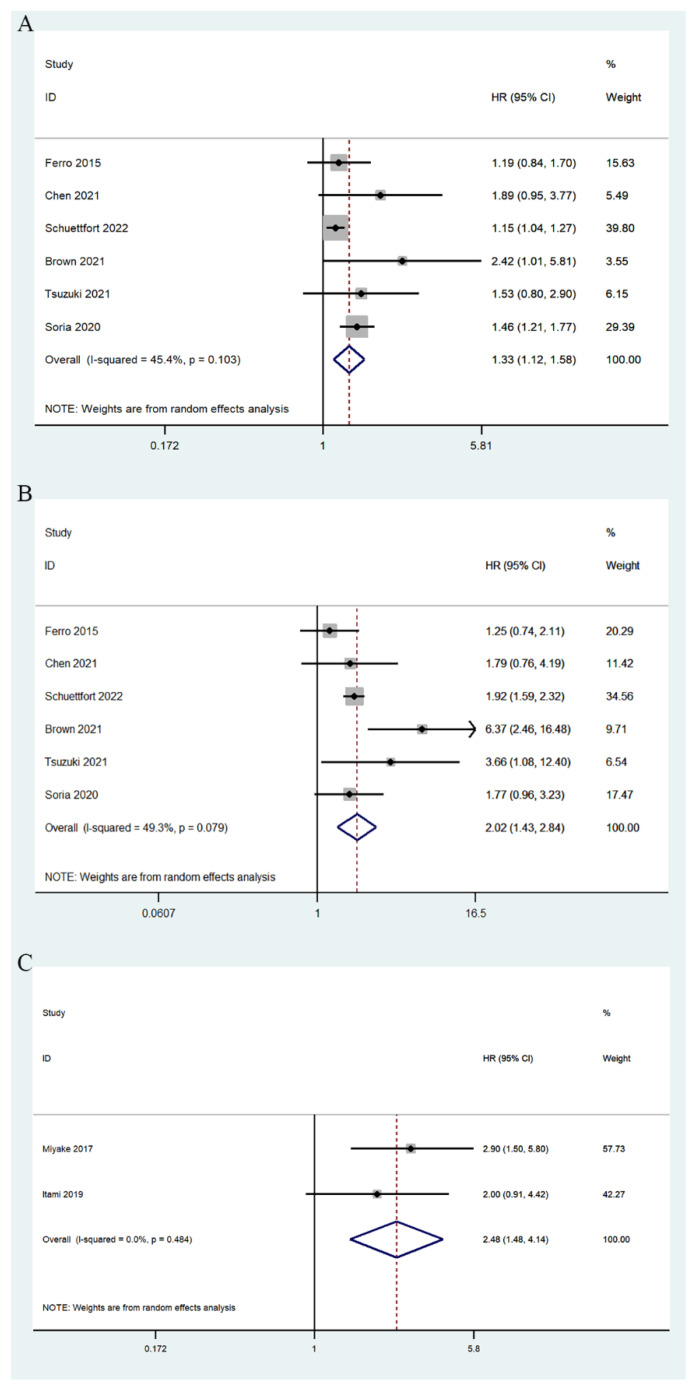
Forest plots of relationship between mGPS and OS in patients with urothelial carcinoma: (**A**) mGPS 1 vs. 0; (**B**) mGPS 2 vs. 0; (**C**) mGPS high vs. low [17,23,26,27,28,29,30,31].

Eight studies involving 8699 patients reported a correlation between mGPS and OS [17,23,26,27,28,29,30,31]. The summary analysis showed that there was a significant association between high pretreatment mGPS and worse survival rates (mGPS 1/0: HR = 1.33, 95% CI 1.12–1.58, *p* = 0.001; mGPS 2/0: HR = 2.02, 95% CI 1.43–2.84, *p* < 0.0001; mGPS high/low: HR = 2.48, 95% CI 1.48–4.14, *p* = 0.001) (Figure 3). At the same time, a subgroup analysis including the tumor type, treatment, ethnicity, and sample size was performed to explore possible sources of heterogeneity. The subgroup analysis showed similar results; high pre-treatment mGPS was significantly associated with poor OS (Table 3).

### 3.4. mGPS and PFS

**Figure 4:**
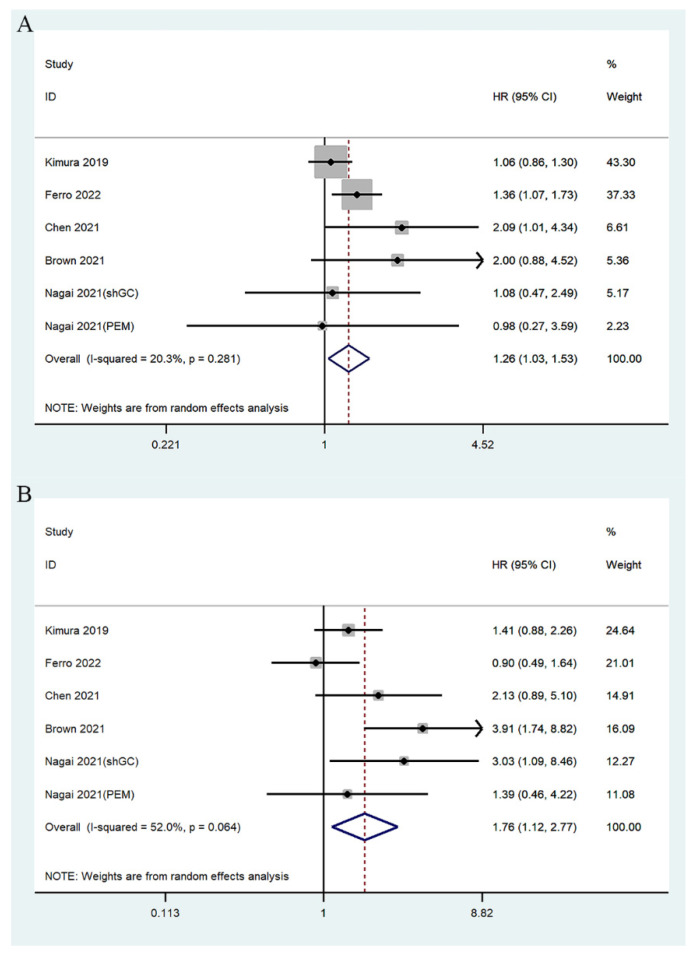
Forest plots of relationship between mGPS and PFS in patients with urothelial carcinoma: (**A**) mGPS 1 vs. 0; (**B**) mGPS 2 vs. 0 [24,29,30,32,33].

Five studies involving 2940 patients reported the relationship between mGPS and PFS [24,29,30,32,33]. The summary analysis showed that high pretreatment mGPS in patients with UC was an independent predictor of PFS (mGPS 1/0: HR = 1.26, 95% CI 1.03–1.53, *p* = 0.021; mGPS 2/0: HR = 1.76, 95% CI 1.12–2.77, *p* = 0.013) (Figure 4).

### 3.5. mGPS and RFS

**Figure 5:**
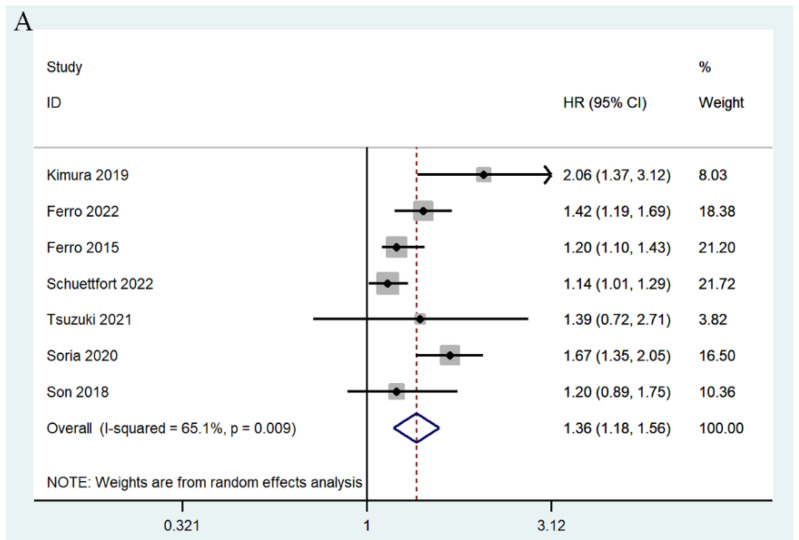

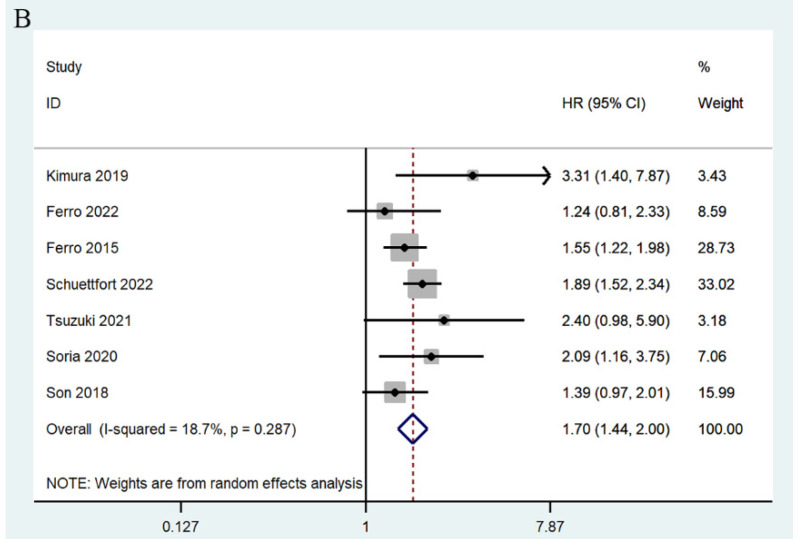
Forest plots of relationship between mGPS and RFS in patients with urothelial carcinoma: (**A**) mGPS 1 vs. 0; (**B**) mGPS 2 vs. 0 [17,24,25,27,28,31,33].

Seven studies involving 11,752 patients reported an association between mGPS and RFS [17,24,25,27,28,31,33]. The summary analysis showed that high pretreatment mGPS in patients with UC was an independent predictor of RFS (mGPS 1/0: HR = 1.36, 95% CI 1.18–1.56, *p* < 0.0001; mGPS 2/0: HR = 1.70, 95% CI 1.44–2.000, *p* < 0.0001) (Figure 5).

### 3.6. mGPS and CSS

**Figure 6:**
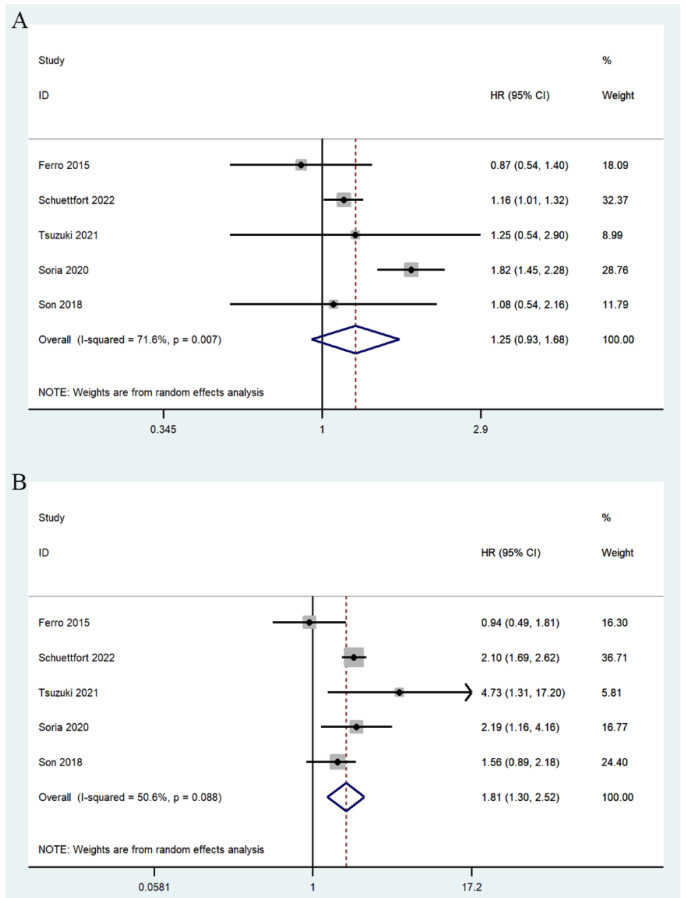

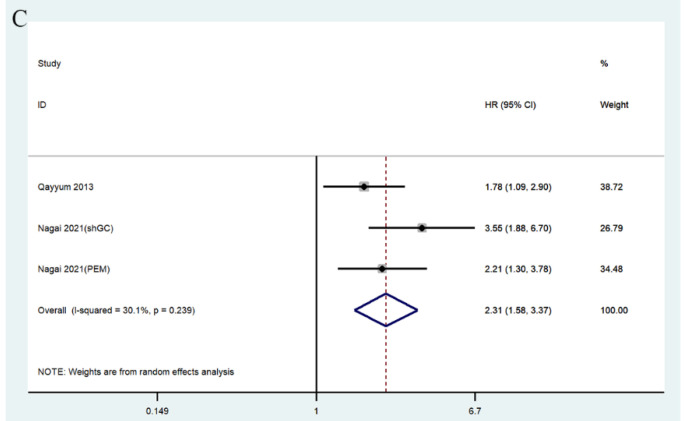
Forest plots of relationship between mGPS and CSS in patients with urothelial carcinoma: (**A**) mGPS 1 vs. 0; (**B**) mGPS 2 vs. 0; (**C**) mGPS high vs. low [17,22,25,27,28,31,32].

Seven studies involving 9484 patients reported the relationship between mGPS and CSS [17,22,25,27,28,31,32]. The summary analysis showed that the association was not statistically significant between a score of 1 and poor CSS (mGPS 1/0: HR = 1.25, 95% CI 0.93–1.68, *p* = 0.133) (Figure 6A), but there was a significant association between high score and poor CSS before treatment (mGPS 2/0: HR = 1.81, 95% CI 1.30–2.52, *p* < 0.0001; mGPS high/low: HR = 2.31, 95% CI 1.58–3.37, *p* < 0.0001) (Figure 6B,C).

### 3.7. Sensitivity Analysis

A sensitivity analysis was conducted to assess the reliability of the merged OS, PFS, RFS, and CSS HRs (Figures S1–S4). The leave-one-out test showed that overall HR estimates of these survival results did not change significantly, indicating that the meta-analysis results were relatively stable and reliable.

## 4. Discussion

This meta-analysis summarized all eligible studies, including 12,524 patients for the first time to evaluate the prognostic value of the mGPS in patients with UC. The results showed that a higher mGPS was closely related to lower survival (OS, PFS, RFS, and CSS). In view of the heterogeneity between studies, we conducted a subgroup analysis of OS based on the tumor type, treatment, ethnicity, and sample size. Our results show that the mGPS can be used as an independent predictor of the prognosis of UC.

The prognosis of patients with UC depends on the characteristics of the patient and the tumor. The transurethral resection of bladder tumors and postoperative adjuvant therapy are the main treatment methods for patients with non–muscle-invasive BC (NMIBC) [35]. In a postoperative study of Ta low-grade UC of the bladder, Mastroianni et al. found that gender and the European Organization for Research and Treatment of Cancer (EORTC) risk group are independent predictors of cancer recurrence, while the absence of the detrusor muscle does not affect RFS [36]. Cicione et al. showed that the ultrasound detection of the bladder detrusor wall’s thickness increases the risk of recurrence and progression in patients with NMIBC [37]. Radical cystectomy is the standard treatment for localized muscle-invasive BC [38]. Even for elderly patients over 80 years old, the frailty index helps guide clinical decision making and, thus, improves patient prognosis [39,40]. Radical nephroureterectomy plus bladder cuff resection is the standard treatment for patients with high-risk non-metastatic UTUC [2]. To date, the most important histopathological prognostic variables are tumor stage and lymph node status after radical resections [38]. However, the surgical approach or completion with intracorporeal urinary diversion does not affect the survival of patients with UC [41,42].

Although clinical features such as tumor stage and lymph node status are the most important factors affecting prognosis, the prognosis of patients with similar clinical manifestations is different, which requires more controllable indicators to predict the prognosis of patients. Cancer-related inflammation is the seventh hallmark of cancer, and inflammatory cytokines produced by tumors and related host cells affect tumor characteristics, including survival, proliferation, angiogenesis, and the metastasis of malignant cells [43]. Therefore, in addition to individual patient and tumor characteristics, clinical markers such as the neutrophil-to-lymphocyte ratio, insulin-like growth factor-I and its binding protein, insulin-like growth factor-I binding protein-2 and -3, the platelet-lymphocyte ratio [44,45,46], molecular markers such as molecular subtypes, circulating tumor cells, and DNA damage repair-gene defects are also used to predict the prognosis of UC [47,48,49].

However, studies have shown that systemic inflammatory responses and nutritional deficiencies may play crucial roles in the development and progression of human cancer. CRP is a marker of systemic inflammation and has been used to determine the prognosis and predict the clinical results of cancer patients [50]. Serum albumin is one of the most common nutritional indicators and is often used to assess nutritional statuses, disease severity, disease progression, and prognosis [51]. Hypoproteinemia is often associated with nutritional deficiencies, poor working conditions, and weight loss, and it negatively impacts the prognosis of cancer patients [52]. The GPS, first described by Forrest et al. [53,54], combined serum albumin and CRP levels and could provide more comprehensive and accurate prognostic information than using albumin or CRP alone, and it could simultaneously assess the patient’s inflammation and nutritional status. Further studies by McMillan et al. showed that the CSS of patients with simple hypoalbuminemia was significantly higher than that of patients with elevated CRP levels. Therefore, the GPS was modified, and only one point was assigned to an elevated CRP concentration [55]. Proctor also found that low albumin level was unrelated to the low survival rate of some cancers (gastroesophageal, bladder, prostate, gynecological, renal, colorectal, neck, hepatopancreaticobiliary, and head) in a larger cohort study, indicating that the mGPS had greater consistency and a better prognostic value than that of the GPS [56].

Ferro et al. study found that mGPS is associated with smoking habits, high tumor grade, and concomitant carcinoma in situ in UC [17]. Qayyum et al. also showed that high mGPS is directly related to tumor stage, grade, and progression in UC [22]. Several meta-analyses have also discussed the prognostic role of mGPS in solid tumors. Jiang summarized and analyzed the data of 72 studies and found that mGPS had a medium predictive ability for OS, DFS, and CSS in esophageal cancer [57]. Wu summarized 25 studies that found that an elevated mGPS before treatment was a sign of poor prognosis in patients with pancreatic cancer [58]. In addition, mGPS is obtained from blood samples, has low cost and high efficiency, is easy to obtain and promote, and can be obtained before treatment. Therefore, we searched the relevant literature and performed a meta-analysis. A summary analysis of 13 studies determined that the higher the mGPS score, the worse the survival results (OS, PFS, RFS, and CSS) of patients. We also confirmed that mGPS had a predictive effect on OS, PFS, RFS, and CSS in patients with UC. These analyses indicate that a high mGPS is closely related to low survival rate in patients with UC. Pre-treatment mGPS is a powerful prognostic marker for patients with UC, which helps guide clinical practice and make appropriate treatment decisions.

Although this analysis systematically analyzed the predictive value of the mGPS for patients with UC before treatment, there are still some limitations. All included studies were retrospective studies with increasing bias. The patients included in the study had substantial differences in pathological staging and treatment methods, which may have led to different survival results and increased heterogeneity among the studies. In addition, in the included studies, there is a great difference in postoperative adjuvant therapy, which is difficult to analyze. The reason is that the adjuvant therapy was mostly determined by the therapist according to guideline recommendations. Moreover, postoperative management strategies vary in different regions and centers, most of the research cycles were long, and the adjuvant therapy guidelines were constantly revised over time. To overcome these limitations, further multicenter prospective studies with larger sample sizes are needed.

## 5. Conclusions

This meta-analysis confirms the close relationship between a high mGPS and the poor prognosis of UC. The mGPS is a simple, effective, and practical prognostic biomarker that can provide an important reference for clinical decision making in the treatment of UC. However, large-scale prospective studies are required before widespread clinical applications.

## Figures and Tables

**Table 1:** The modified Glasgow Prognostic Scores.

mGPS	Points Allocated
CRP ≤ 10 mg/L and albumin ≥ 35 g/L	0
CRP > 10 mg/L	1
CRP > 10 mg/L and albumin < 35 g/L	2

mGPS = modified Glasgow Prognostic Score; CRP = C-reactive protein.

**Table 2:** Baseline characteristics of include studies and methodological assessment.

Author	Year	Country	Study Design	Tumor Type	mGPS Group	Treatment	Sample Size	Age (Years)	Analysis Method	Survival Analysis	Follow-Up (Months)	Quality Score
Qayyum et al. [22]	2013	United Kingdom	Retrospective	BC	High/low	Non-Surgery	68	Median72 (range, 43–93)	Multivariate	CSS	Median47 (range, 1.2–201)	8
Ferro et al. [17]	2015	Italy	Retrospective	BC	0/1/2	RC	1037	Median70 (range, 42–88)	Multivariate	RFS/OS/CSS	Median22 (range, 3–60)	9
Miyake et al. [23]	2017	Japan	Retrospective	BC	High/low	RC	117	Median72 (IQR, 61–77)	Multivariate	OS	Median22 (IQR, 10–64)	8
Son et al. [25]	2018	Korea	Retrospective	UTUC	0/1/2	RNU	1137	Median69 (IQR, 61–74)	Multivariate	RFS/CSS	Median39.1 (IQR, 18.3–63.8)	9
Kimura et al. [24]	2019	Austria	Retrospective	BC	0/1/2	TURB	1096	Median67 (IQR, 58–74)	Multivariate	PFS/RFS	Median64.8 (IQR, 26.5–110.9)	8
Itami et al. [26]	2019	Japan	Retrospective	UTUC	High/low	RNU	125	Median72 (range, 38–90)	Multivariate	OS	Median51 (range, 6–227)	8
Soria et al. [27]	2020	Italy	Retrospective	UTUC	0/1/2	RNU	2492	Median69 (IQR, 61–76)	Multivariate	RFS/CSS/OS	Median45 (IQR, 20–81)	9
Nagai et al. [32]	2021	Japan	Retrospective	mUC	High/low	shGC	68	-	Multivariate	CSS/PFS	-	6
Nagai et al. [32]	2021	Japan	Retrospective	mUC	High/low	PEM	74	-	Multivariate	CSS/PFS	-	6
Tsuzuki et al. [28]	2021	Japan	Retrospective	UTUC	0/1/2	RNU	273	Median71 (IQR, 63–77)	Multivariate	RFS/CSS/OS	Median36.1	8
Brown et al. [29]	2021	USA	Retrospective	mUC	0/1/2	ICIs	53	Median70 (range, 32–86)	Multivariate	PFS/OS	Median27.1	8
Chen et al. [30]	2021	China	Retrospective	BC	0/1/2	RC	267	-	Univariate	PFS/OS	-	8
Ferro et al. [33]	2022	Italy	Retrospective	BC	0/1/2	BCG	1382	Mean69.87 (IQR, 60.16–79.58)	Multivariate	PFS/RFS	Median44 (IQR, 36–58)	9
Schuettfort et al. [31]	2022	Austria	Retrospective	BC	0/1/2	RC	4335	Median67 (IQR, 60–73)	Multivariate	RFS/OS/CSS	Median41 (IQR, 18.3–60.8)	9

BC = bladder cancer; UTUC = upper urinary tract urothelial carcinoma; mGPS = modified Glasgow Prognostic Score; mUC = metastatic urothelial cell carcinoma; ICIs = immune checkpoint inhibitors; OS = overall survival; PFS = progression-free survival; RFS = recurrence-free survival; CSS = cancer-specific survival; RC = radical cystectomy; BCG = Bacillus Calmette–Guerin; TURB = transurethral resection of bladder; shGC = short hydration gemcitabine/cisplatin; PEM = pembrolizumab; RNU = remains radical nephroureterectomy; IQR = Interquartile Range.

**Table 3:** Subgroup analyses of OS.

Outcome	Variable	No. of Studies	Model	HR (95% CI)	*p*	Heterogeneity	
						I^2^ (%)	*p*
OS (1/0)	All	6	Random	1.33 (1.12, 1.58)	0.001	45.4	0.103
Ethnicity	Caucasian	4	Random	1.29 (1.07, 1.56)	0.008	58.0	0.068
	Asian	2	Random	1.69 (1.06, 2.70)	0.029	0.0	0.657
Tumor type	BC	3	Random	1.16 (1.06, 1.28)	0.002	0.0	0.368
	mUC	1	-	2.42 (1.01, 5.80)	0.048	-	-
	UTUC	2	Random	1.47 (1.22, 1.76)	0.000	0.0	0.891
Sample size	≤1500	4	Random	1.44 (1.09, 1.90)	0.009	3.8	0.374
	>1500	2	Random	1.28 (1.01, 1.61)	0.004	78.9	0.029
Treatment	Surgery	5	Random	1.29 (1.10, 1.51)	0.001	41.5	0.145
	Non-Surgery	1	-	2.42 (1.01, 5.80)	0.048	-	-
OS (2/0)	All	6	Random	2.02 (1.43, 2.84)	0.000	49.3	0.079
Ethnicity	Caucasian	4	Random	1.99 (1.29, 3.06)	0.002	65.6	0.033
	Asian	2	Random	2.26 (1.13, 4.54)	0.022	49.3	0.079
Tumor type	BC	3	Random	1.78 (1.42, 2.23)	0.000	12.4	0.319
	mUC	1	-	6.37 (2.46, 16.49)	0.000	-	-
	UTUC	2	Random	2.08 (1.15, 3.77)	0.015	8.4	0.296
Sample size	≤1500	4	Random	2.47 (1.15, 5.31)	0.020	69.3	0.021
	>1500	2	Random	1.91 (1.59, 2.28)	0.000	0.0	0.802
Treatment	Surgery	5	Random	1.85 (1.56, 2.18)	0.000	0.0	0.474
	Non-Surgery	1	-	6.37 (2.46, 16.49)	0.000	-	-

OS = overall survival; BC = bladder cancer; UTUC = upper urinary tract urothelial carcinoma; mUC = metastatic urothelial cell carcinoma; HR = hazard ratio; CI = confidence interval.

## Data Availability

Not applicable.

## Supplementary Materials

The following supporting information can be downloaded at: https://www.mdpi.com/article/10.3390/jcm11216261/s1, Figure S1. Sensitivity analysis of the effect of modified Glasgow prognostic score on overall survival in urothelial carcinoma: (A) mGPS 1 vs. 0; (B) mGPS 2 vs. 0; (C) mGPS high vs. low. Figure S2. Sensitivity analysis of the effect of modified Glasgow prognostic score on progression free survival in urothelial carcinoma: (A) mGPS 1 vs. 0; (B) mGPS 2 vs. 0. Figure S3. Sensitivity analysis of the effect of modified Glasgow prognostic score on recurrence free survival in urothelial carcinoma: (A) mGPS 1 vs. 0; (B) mGPS 2 vs. 0. Figure S4. Sensitivity analysis of the effect of modified Glasgow prognostic score on cancer specific survival in urothelial carcinoma: (A) mGPS 1 vs. 0; (B) mGPS 2 vs. 0; (C) mGPS high vs. low.
