# Supplementary material for: Prognostic Utility of the Modified Glasgow Prognostic Score in Urothelial Carcinoma: Outcomes from a Pooled Analysis
Source: J Clin Med. 2022 Oct 24;11(21):6261. doi: 10.3390/jcm11216261 (PMC9655933; doi:10.3390/jcm11216261)
Supplement: Supplementary file 1 [file jcm-11-06261-s001.zip › jcm-1951798-supplementary.pdf]

Figure S1. Sensitivity analysis of the effect of modified Glasgow Prognostic Score on overall survival in urothelial carcinoma: (**A**) mGPS 1 vs. 0; (**B**) mGPS 2 vs. 0; (**C**) mGPS high vs. low [17,23,26–31].

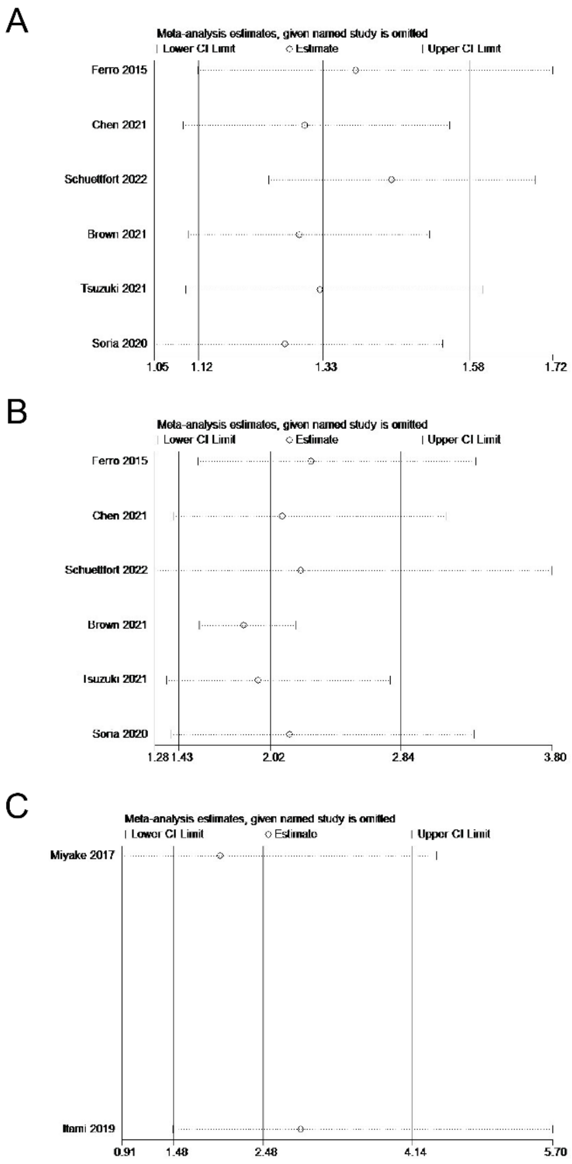

Figure S2. Sensitivity analysis of the effect of modified Glasgow Prognostic Score on progression free survival in urothelial carcinoma: (**A**) mGPS 1 vs. 0; (**B**) mGPS 2 vs. 0 [24,29,30,32,33].

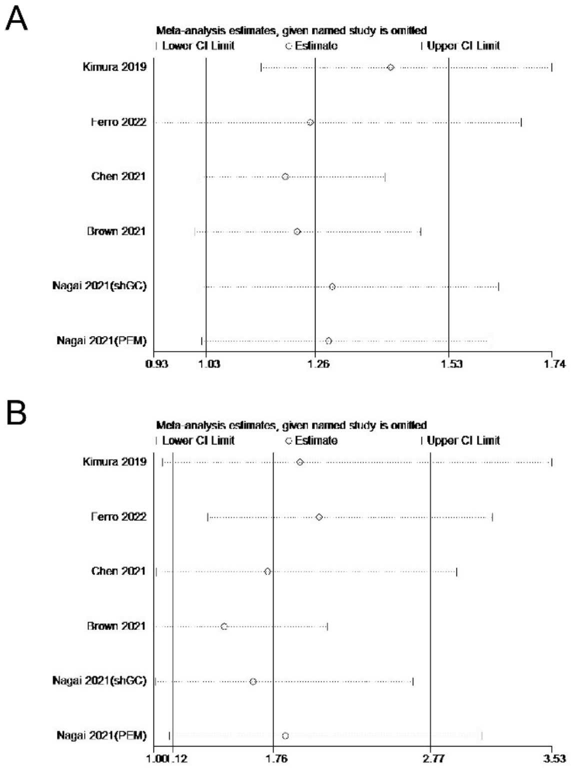

Figure S3. Sensitivity analysis of the effect of modified Glasgow Prognostic Score on recurrence free survival in urothelial carcinoma: (**A**) mGPS 1 vs. 0; (**B**) mGPS 2 vs. 0 [17,24,25,27,28,31,33].

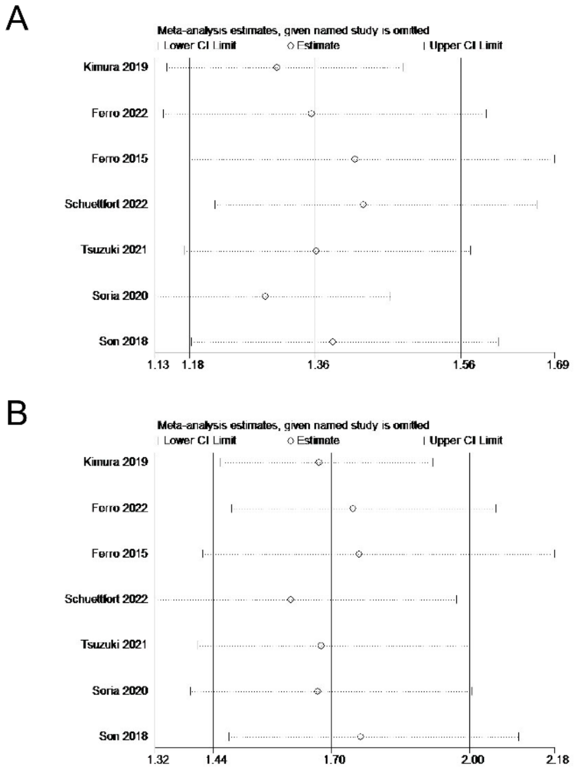

Figure S4. Sensitivity analysis of the effect of modified Glasgow Prognostic Score on cancer specific survival in urothelial carcinoma: (**A**) mGPS 1 vs. 0; (**B**) mGPS 2 vs. 0; (**C**) mGPS high vs. low [17,22,25,27,28,31,32].

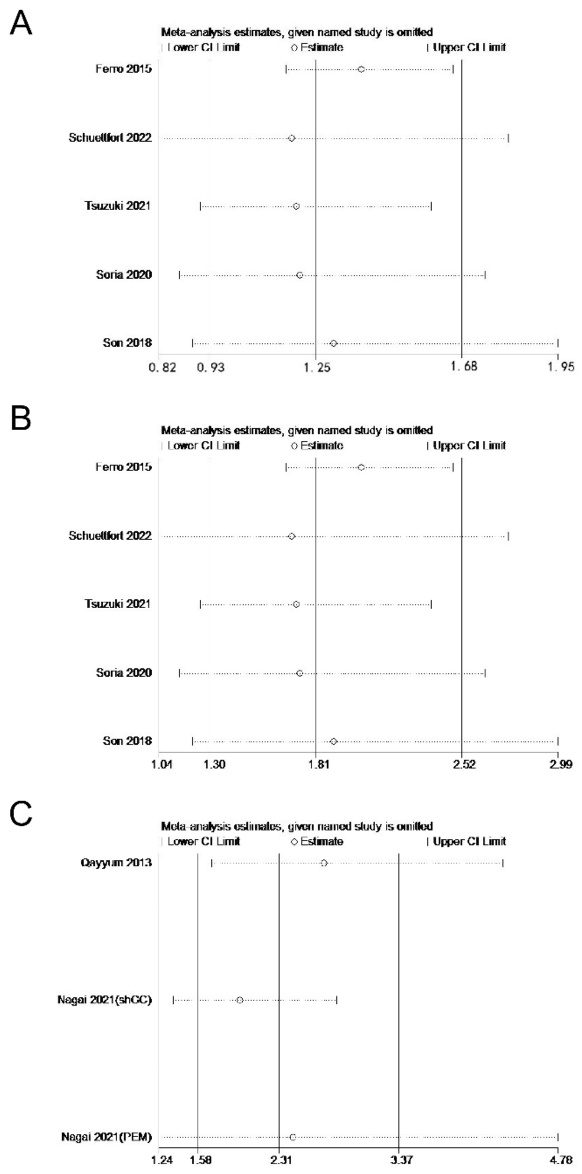

Reference:

17. Ferro, M.; De Cobelli, O.; Buonerba, C.; Di Lorenzo, G.; Capece, M.; Bruzzese, D.; Autorino, R.; Bottero, D.; Cioffi, A.; Matei, D.V.; et al. Modified Glasgow Prognostic Score is Associated with Risk of Recurrence in Bladder Cancer Patients After Radical Cystectomy. *Medicine* **2015**, *94*, e1861.

22. Qayyum, T.; McArdle, P.; Hilmy, M.; Going, J.; Orange, C.; Seywright, M.; Horgan, P.; Underwood, M.; Edwards, J. A Prospective Study of the Role of Inflammation in Bladder Cancer. *Curr. Urol.* **2013**, *6*, 189–193.

23. Miyake, M.; Morizawa, Y.; Hori, S.; Marugami, N.; Iida, K.; Ohnishi, K.; Gotoh, D.; Tatsumi, Y.; Nakai, Y.; Inoue, T.; et al. Integrative Assessment of Pretreatment Inflammation-, Nutrition-, and Muscle-Based Prognostic Markers in Patients with Muscle-Invasive Bladder Cancer Undergoing Radical Cystectomy. *Oncology* **2017**, *93*, 259–269.

24. Kimura, S.; D′ Andrea, D.; Soria, F.; Foerster, B.; Abufaraj, M.; Vartolomei, M.D.; Iwata, T.; Karakiewicz, P.I.; Rink, M.; Gust, K.M.; et al. Prognostic value of modified Glasgow Prognostic Score in non–muscle-invasive bladder cancer. *Urol. Oncol. Semin. Orig. Investig.* **2019**, *37*, e119–e179.

25. Son, S.; Hwang, E.-C.; Jung, S.-I.; Kwon, D.-D.; Choi, S.-H.; Kwon, T.-G.; Noh, J.-H.; Kim, M.-K.; Seo, I.-Y.; Kim, C.-S.; et al. Prognostic value of preoperative systemic inflammation markers in localized upper tract urothelial cell carcinoma: A large, multicenter cohort analysis. *Minerva Urol. Nephrol.* **2018**, *70*, 300–309.

26. Itami, Y.; Miyake, M.; Tatsumi, Y.; Gotoh, D.; Hori, S.; Morizawa, Y.; Iida, K.; Ohnishi, K.; Nakai, Y.; Inoue, T.; et al. Preoperative predictive factors focused on inflammation-, nutrition-, and muscle-status in patients with upper urinary tract urothelial carcinoma undergoing nephroureterectomy. *Int. J. Clin. Oncol.* **2019**, *24*, 533–545.

27. Soria, F.; Giordano, A.; D'Andrea, D.; Moschini, M.; Rouprêt, M.; Margulis, V.; Karakiewicz, P.I.; Briganti, A.; Bensalah, K.; Mathieu, R.; et al. Prognostic value of the systemic inflammation modified Glasgow prognostic score in patients with upper tract urothelial carcinoma (UTUC) treated with radical nephroureterectomy: Results from a large multicenter international collaboration. *Urol. Oncol. Semin. Orig. Investig.* **2020**, *38*, e611–e602.

28. Tsuzuki, S.; Kimura, S.; Fukuokaya, W.; Yanagisawa, T.; Hata, K.; Miki, J.; Kimura, T.; Abe, H.; Egawa, S. Modified Glasgow prognostic score is a pre-surgical prognostic marker of disease mortality in upper urinary tract urothelial carcinoma. *Jpn. J. Clin. Oncol.* **2021**, *51*, 138–144.

29. Brown, J.T.; Liu, Y.; Shabto, J.M.; Martini, D.J.; Ravindranathan, D.; Hitron, E.E.; Russler, G.A.; Caulfield, S.; Yantorni, L.B.; Joshi, S.S.; et al. Baseline Modified Glasgow Prognostic Score Associated with Survival in Metastatic Urothelial Carcinoma Treated with Immune Checkpoint Inhibitors. *Oncologist* **2021**, *26*, 397–405.

30. Chen, J.; Hao, L.; Zhang, S.; Zhang, Y.; Dong, B.; Zhang, Q.; Han, C. Preoperative Fibrinogen–Albumin Ratio, Potential Prognostic Factors for Bladder Cancer Patients Undergoing Radical Cystectomy: A Two-Center Study. *Cancer Manag. Res.* **2021**, *13*, 3181–3192.

31. Schuettfort, V.M.; Gust, K.; D'Andrea, D.; Quhal, F.; Mostafaei, H.; Laukhtina, E.; Mori, K.; Rink, M.; Abufaraj, M.; Karakiewicz, P.I.; et al. Impact of the preoperative modified Glasgow Prognostic Score on disease outcome after radical cystectomy for urothelial carcinoma of the bladder. *Minerva Urol. Nephrol.* **2022**, 74, 302–312.

32. Nagai, T.; Naiki, T.; Isobe, T.; Sugiyama, Y.; Etani, T.; Iida, K.; Nozaki, S.; Noda, Y.; Shimizu, N.; Tasaki, Y.; et al. Modified Glasgow Prognostic Score 2 as a Prognostic Marker in Patients With Metastatic Urothelial Carcinoma. *Vivo* **2021**, *35*, 2793–2800.

33. Ferro, M.; Tătaru, O.S.; Musi, G.; Lucarelli, G.; Abu Farhan, A.R.; Cantiello, F.; Damiano, R.; Hurle, R.; Contieri, R.; Busetto, G.M.; et al. Modified Glasgow Prognostic Score as a Predictor of Recurrence in Patients with High Grade Non-Muscle Invasive Bladder Cancer Undergoing Intravesical Bacillus Calmette–Guerin Immunotherapy. *Diagnostics* **2022**, *12*, 586.
